#  Pure Esophageal Atresia without a Gap: An Unusual Variant 

**Published:** 2016-04-10

**Authors:** Dhiraj Parihar, Yogender Singh Kadian, Kamal Nain Rattan

**Affiliations:** 1 Department of Paediatric Surgery, B P S Government Medical College For Women Khanpur Kalan, India; 2 Department of Paediatric Surgery, PT B D Sharma PGIMS Rohtak, India

**Dear Sir**

Pure esophageal atresia (EA) constitutes around 6-7% of all congenital anomalies of oesophagus and is associated with a long gap between the upper and lower ends which make primary repair almost impossible [1]. But in literature there are isolated reports where the two esophageal ends were joined either by a membrane or when the upper pouch is longer than the usual [2, 3].

A (2.4 Kg) female full-term baby admitted with respiratory distress and frothing from the mouth since birth. The infantogram with the nasogastric tube in situ revealed the position of the tube was at T7-T8 vertebral level and there was no gas in the abdomen (Fig. 1). There was no other associated anomaly. At operation, the upper and lower pouches were almost in continuity but there was a 1cm long atretic segment between them, without tracheo-esophageal fistula (Fig. 2). The atretic segment was excised and primary anastomosis was done between the two ends with nasogastric tube in place. The patient was put on ventilator in postoperative period because of the pneumonitis. The chest tube was removed on 5th day and the patient was weaned off the ventilator; the nasogastric tube was removed on 10th day. The histopathological examination of atretic segment show fibromuscular layer without mucosa. 

Kluth has described ten types of EA in his atlas; pure atresia is classified as type II in which the proximal and distal segments are atretic without a tracheo-esophageal fistula [4]. In this condition the gap between two pouches makes primary anastomosis impossible and management options remain either delayed primary repair or oesophageal substitution. However variation to this typical pure EA is also reported [2]. Loosbroek et al described a new type of pure EA that included double membranes with 2cm gap between them [3]. The present case resembles that of pure atresia reported by Lall [2] as well as by Loosbroek [3]; where the two ends were joined together by a membranous structure but was managed by thoracotomy and primary repair. At times it is challenging to make a preoperative differentiation between a pure EA or a blocked distal fistula [5]. Preoperative endoscopic examination or a contrast study via gastrostomy ascertains the length of distal esophagus as well as the status of fistula [5].

**Figure:**
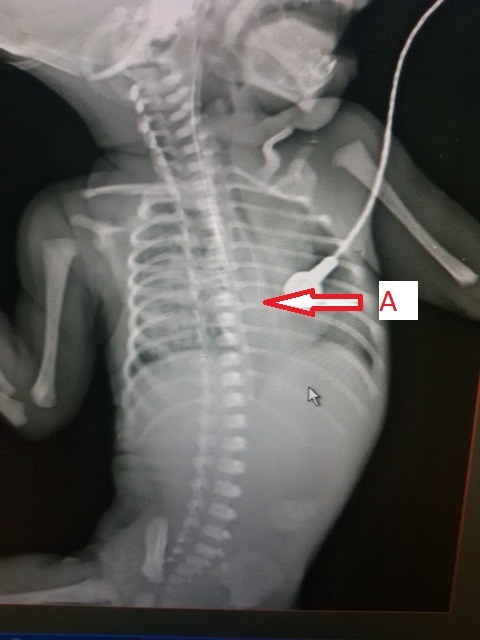
Figure 1: The X-ray with nasogastric tube in situ showing the level of upper pouch at the level of T7–T8 vertebrae (A) and gasless abdomen

**Figure:**
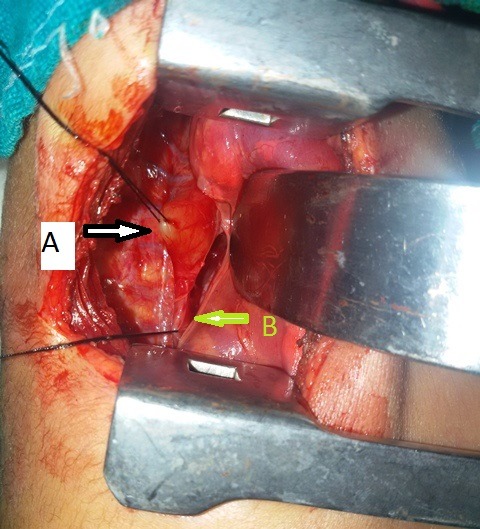
Figure 2: The operative photograph showing two ends of the esophagus (A-upper end and B- lower end) with intervening membrane.

## Footnotes

**Source of Support:** Nil

**Conflict of Interest:** None
